# The Global research of protein post-translational modifications in the cancer field: A bibliometric and visualized study

**DOI:** 10.3389/fonc.2022.978464

**Published:** 2022-11-03

**Authors:** Ruixia Ma, Meigui Zhang, Jiahui Xi, Jing Li, Yinxia Ma, Binxiao Han, Tuanjie Che, Zhihui Ma, Jinhui Tian, Zhongtian Bai

**Affiliations:** ^1^ The First School of Clinical Medicine; The First Hospital of Lanzhou University, Lanzhou University, Lanzhou, China; ^2^ The Second Clinical Medical School, Lanzhou University, Lanzhou, China; ^3^ Gansu Institute of Medical Information, Institute of Gansu Medical Science Research, Lanzhou, China; ^4^ Key Laboratory of Functional Genomic and Molecular Diagnosis of Gansu Province, Lanzhou, China; ^5^ School of Mathematics and Statistics, Lanzhou University, Lanzhou, China; ^6^ Evidence-Based Medicine Center School of Basic Medical Sciences, Lanzhou University, Lanzhou, China; ^7^ Key Laboratory Biotherapy and Regenerative Medicine, The First Hospital of Lanzhou University, Lanzhou, China

**Keywords:** post-translational modification, tumor, citespace, VOSviewer, histcite, knowledge graph, bibliometrics

## Abstract

**Objectives:**

Protein post-translational modifications (PTMs) are closely associated with tumorigenesis, targeting PTMs of key proteins might be the focus of antitumor drug discovery. This study aimed to analyze the research progress on protein PTMs in tumorigenesis by performing qualitative and quantitative evaluations.

**Methods:**

The Web of Science Core Collection was selected as the database, and Science Citation Index Expanded was selected as the citation index. Visualization tools such as VOSviewer, CiteSpace, HistCite, and Online Analysis Platform of Bibliometrics were used to deeply explore the information of the retrieved research papers and analyze them in terms of research trends and main aspects of research.

**Results:**

The search yielded 3777 relevant articles. The results showed that the total number of PTMs related papers in cancer field has been increasing annually, with the largest number of papers published in the United States of America. The co-word cluster analysis showed that the research on PTMs and tumorigenesis was primarily focused on the following four areas, mechanism, histone, P53, key Technologies. Tumor metabolism, autophagy, and protein-protein interaction, histone ubiquitination have become new research topics.

**Conclusion:**

This study provides an important reference for the research direction and selection of topics of interest in the PTMs of cancer field.

## 1 Introduction

Protein post-translational modifications (PTMs) represent important regulatory modalities of protein function; they alter the charge properties, hydrophilicity/hydrophobicity, and conformation of proteins. PTMs are extremely diverse, primarily being associated with processes such as phosphorylation, ubiquitination, methylation, and acetylation. Currently, several modifications, such as succinylation, lactylation, crotonylation, malonylation, and trihydroxybutanylation have been discovered, and their critical roles in disease progression have been investigated. PTMs represent key processes within signal transduction of phosphate, acetyl, and glycosyl groups, which are conducted from one protein to another. As most PTMs are reversible, they are used as ‘switches’ in normal cells to determine the cell states (quiescent or active), thereby rapidly and tightly regulating cell proliferation (1). To date, 461 unique types of modifications have been identified, including phosphorylation, acetylation, ubiquitination, and ubiquitination (2). These modifications regulate the development of various diseases in the nervous, endocrine, and cardiovascular systems by altering the activity, intracellular distribution, and interactions of the targeted proteins (3–5).

In the early stages, bibliometric analysis was primarily used by researchers in disciplines such as intelligence, library science, and archives. However, with the advancement of research and the emergence of cross-disciplinary approaches, this analysis is now widely used in numerous fields within the medical discipline. For instance, Huang et al. used CiteSpace and VOSviewer software to analyze emerging trends and research foci in the gastrointestinal microbiome, which showed that gut microbiota, inflammatory bowel disease, probiotics, irritable bowel disease, and obesity are hotspots in gut microbiome research, whereas bile acids, obesity, and myxobacteria represent new research foci (6). Furthermore, Wu et al. conducted a knowledge graph analysis and visualization of applied Artificial Intelligence techniques in the new coronary pneumonia epidemic, presenting researchers and practitioners with perspectives on the challenges and limitations of Artificial Intelligence applications in COVID-19 and facilitating research on Artificial Intelligence applied to COVID-19 (7). A different research group mapped knowledge structures and thematic trends in rheumatoid arthritis osteoporosis, providing a field with potential research frontiers and hot directions (8). Similarly, tumor PTMs have received considerable attention in recent years, and the characteristics as well as the regulatory mechanisms of PTMs have been shown to exhibit potential roles in tumorigenesis, prognosis, and drug development. However, the complicated nature of the literature discussing PTMs in cancer field can prove to be confusing even for experts and scholars when presented in the absence of clear future directions. Bibliometrics analysis provides a timely, visual, relatively objective way to a create a representative sample of articles, journals and publishers, track the development and explore the knowledge structure in a specific knowledge field (9).

In this study, a bibliometric analysis of international research on PTMs in cancer field was conducted using VOSviewer, CiteSpace, and HistCite and Online Analysis Platform of Bibliometrics visual analysis software tools based on the status of PTMs related papers in cancer field deposited in the Web of Science Core Collection (WOSCC) Science Citation Index Expanded (SCI-EXPANDED) database.

## 2 Materials and methods

### 2.1 Data sources

Web of Science is considered to be the most prominent database of scientific publications on many research topics. It provides several databases covering interdisciplinary research and explores the professional areas of many disciplines (10). The cluster analysis of literature co-citation can explore the common topics of similar documents, which is one of the most powerful functions of CiteSpace and the most important part of bibliometrics analysis. But the co-citation analysis can only analyze the papers searched from WOSCC (11). HistCite also supports only Web of Science databases and is powerless for databases such as Scopus and PubMed. In order to meet the requirements of data analysis, We retrieved articles in the Web of Science Core Collection Science Citation Index Expanded (SCI-Expanded) from inception to April 11^th^, 2022. The retrieval formula is as follows: #1 =(((((((((TS=(Neoplasias)) OR TS=(Neoplasm)) OR TS=(Tumors)) OR TS=(Tumor)) OR TS=(Cancer)) OR TS=(Cancers)) OR TS=(Malignancy)) OR TS=(Malignancies)) OR TS=(Malignant)) OR TS=(Benign). #2=(((TS=(Post Translational Protein Processing)) OR TS=(Post translational Modifications)) OR TS=(Post Translational Protein Modifications)) OR TS=(Post Translational Amino Acid Modification). #3= #1 AND #2.

**Figure 1:**
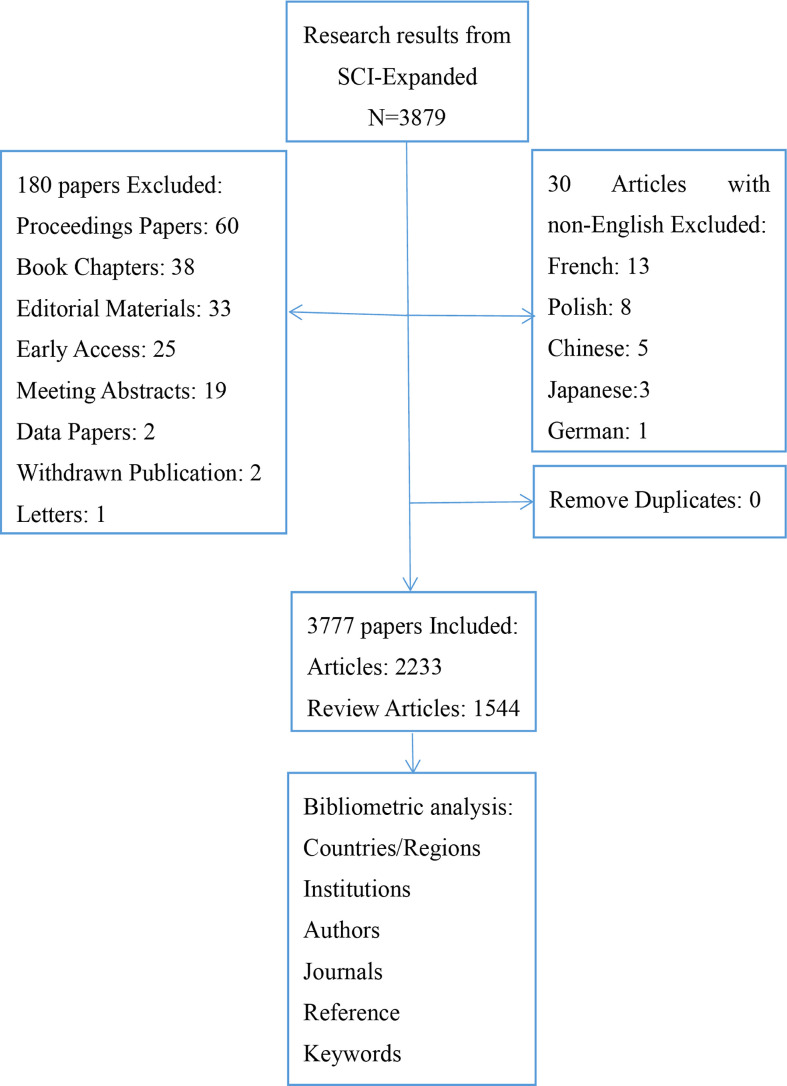
Flow chart of literature inclusion and exclusion. Source: authors with Word.

With #3 searched, we obtained 3879 documents, and following the selection of “English”, “Articles”, and “Reviewer articles,” 3777 articles were included in the analysis. The flow chart of literature screening is shown in **Figure 1**.

### 2.2 Data analysis methods

#### 2.2.1 Analysis of literature publication time and countries/regions

Online Analysis Platform of Bibliometrics (http://bibliometric.com/) was used to analyze trends in the number of papers issued and the annual number of papers issued by each country to determine the literature distribution.

#### 2.2.2 Analysis of the academic connection between countries/regions, institutions, authors and journals

Analyzing the co-citation among countries or regions, institutions and authors to determine the countries, institutions and authors who have made great contributions in this field and their potential cooperative relations;

VOSviewer 1.6.17 software was used to analyze and generate the collaboration between countries/regions, organizations, and a co-citation network diagram for high-yield authors. The specific settings were as follows: type of analysis: co-citation; unit of analysis: cited authors; choice threshold: minimum number of citations of an author was 50. Of the 128,093 authors, 250 met the threshold. For the institutions, type of analysis: co-authorship; unit of analysis: organizations; choice threshold: minimum number of documents of an organization was 20. Of the 3217 organizations, 44 met the threshold. For the countries, type of analysis: co-authorship; unit of analysis: countries; choice threshold: minimum number of documents of a country was 5. Of the 76 countries, 48 met the thresholds. The network diagram consists of nodes and connections, where the nodes represent the country/region,organizations or authors. The same color represents the close cooperation among these countries/regions, organizations or authors. We cleaned the data before analyzing, for instance, in the countries/regions analysis, publications from Taiwan were reclassified to China, and those from England, Scotland, Northern Ireland, and Wales were assigned to the United Kingdom (12).

CiteSpace software V5.8.R3 SE, 64 bits (Drexel University, Philadelphia, PA, USA) was used to conduct the dual map overlay of journals.The dual map overlay of journals aims to reveal the topic distribution of journals. Publications and citations in this field can be described at the disciplinary level.

#### 2.2.3 Knowledge-based analysis of PTMs in the cancer field

Analyzing literature co-citation, citation path analysis, keyword clustering and keyword burst to determine the knowledge base and research hotspots in this field, which is the most critical step in this bibliometric analysis.

HistCite software was used to analyze the citation paths of the first 30 highly cited pieces of literature. The citation paths is to map out the development of this field, lock in the important literature of this research direction, and find some papers with groundbreaking achievements without specified keywords. The numbers in the box represent the chronological order of the documents in all downloaded documents, and the closer to the present, the larger the number; The size of the box in the figure is proportional to the local citation score (LCS) of the literature, that is, the bigger the box, the higher the LCS. Normally, the LCS and global citation score (GCS) were used to evaluate the significance of each article. LCS indicates the number of citations of the document in the current literature set, GCS indicates the number of citations in all the resources in the WOSCC database at the time the document was downloaded. The higher the LCS, the higher the importance of the publication in its field of expertise, and the higher the GCS meant that the publication had attracted worldwide attention, regardless of the reader’s specialty. Therefore, it was believed that LCS had a higher reference value than GCS (13).

CiteSpace software was used to analyze and visualize co-cited references, and citation bursts for keywords. Since co-cited references and keyword burst are mainly used to explore common topics and emerging topics in this field, the author analyzes the literature information in the past ten years from 2011 to 2021 in order to avoid too much literature interfering with the real analysis results. The CiteSpace settings were as follows: time span was from 2011 to 2021, years per slice was “1”, pruning selected “Minimum Spanning Tree and Pruning Sliced Networks”, and selection criteria selected “Top N=50”. Labels chosen by the log-likelihood ratio test method (LLR) are used in the subsequent discussions (14). For keywords burst detection, “Keywords” was chosen for Node Type, and others followed the default. After removing keywords with little significance (like cells, mice, etc.), the top 30 keywords with the strongest citation bursts were identified and presented using Microsoft Excel 2019.

VOSviewer 1.6.17 software was used to analyze and generate a cluster network diagram for the co-occurrence of author keywords. “Author keywords” were selected for cluster analysis, and the frequency of more than or equal to 20 times was set as the high-frequency author keywords, 61 met the threshold. The network diagram consists of nodes and connections, where the nodes represent the keywords. We merged the synonyms such as “dna-damage” and “dna damage” in keyword evolution analysis. See the Additional files “replace” for more details. The connections between the nodes represent collaborative, co-occurring, or co-cited relationships. The size of the nodes reflects the frequency of occurrence of the elements, and the frequency of occurrence is positively correlated with the size of the nodes. The colors of the nodes and lines represent different clusters or years (15, 16).

## 3 Results and discussion

### 3.1 Analysis of literature publication time and countries/regions

**Figure 2:**
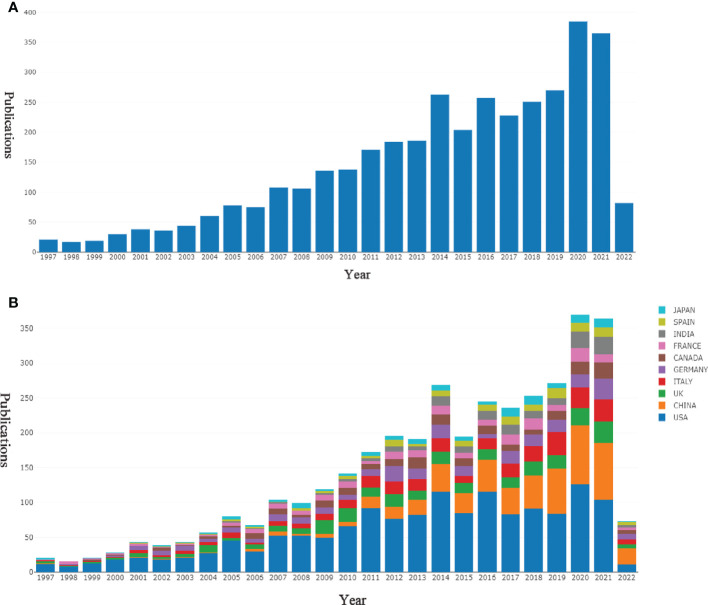
Output of related literature. Source: authors with Online Analysis Platform of Bibliometrics (http://bibliometric.com/). Bar chart reflects number of online articles per year. **(A)** The number of annual publications. **(B)** Growth trends of the top 10 countries/regions in PTMs and cancer field from 1997 to 2021.

As shown in **Figure 2A**, studies related to PTMs in the cancer field included in the WOSCC SCI-EXPANDED database have kept gradually increasing from 1997 to 2021, indicating that the relationship between PTMs and the cancer field will continue to receive international scholarly attention. To find out which countries/regions are leading in this area of research, further analysis of publications from different countries and regions was performed using the bibliometric online analysis platform (http://bibliometric.com/). We found that the US is the pioneer in this field and the number of publications is steadily increasing. Although China initially lagged behind, its annual publication output in this field has grown rapidly (**Figure 2B**).

### 3.2 Analysis of the academic connection between countries/regions, institutions, authors and journals

**Figure 3:**
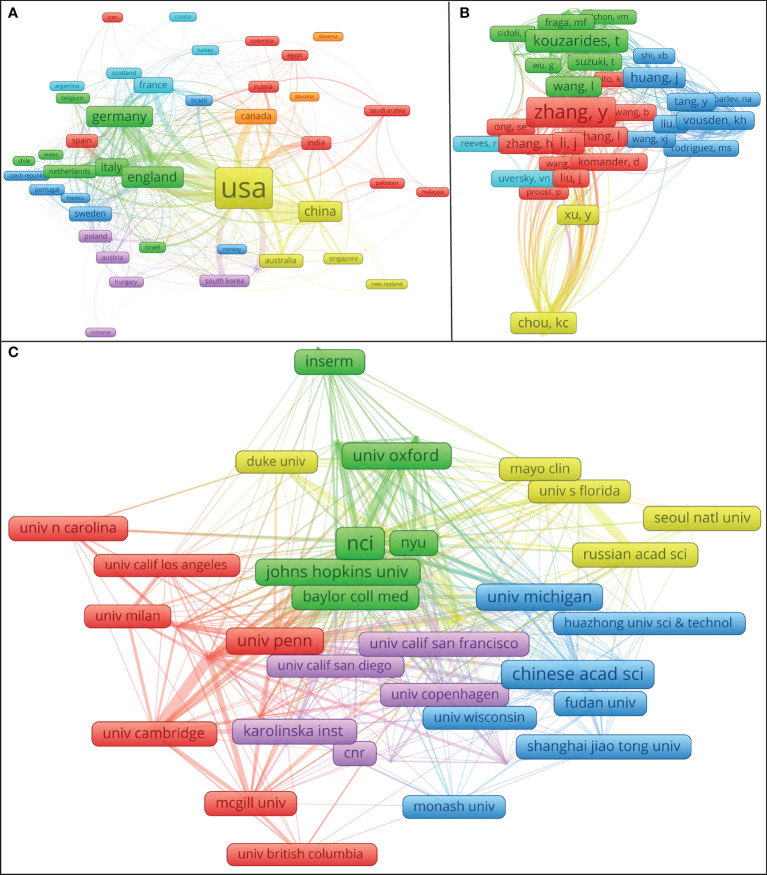
Cooperation maps between countries/regions, institutions, and the authors’ co-citation network visualization map. Source: authors with VOSviewer. **(A)** The coupling of the country to which the literature belongs. Different colors show different clusters. The number of common references cited by the research in countries with the same color is more, the links between them are more frequent, and the research topics are more similar. **(B)** The authors’ co-citation network visualization map. Nodes represent the frequency with which the author has been cited, the thickness of the lines reflects the co-citation strength. The authors with the same color may be a scientific community. When the literature of two authors is cited by the literature of the third author at the same time, it is said that there is a co-citation relationship between the two authors; If the “distance” between two authors is closer, the higher their co-citation frequency is, the closer their academic relationship is. **(C)** The cooperation mapping between institutions. Different colors represent clusters with close cooperation. The thickness of the line between institutions reflects the frequency of the cooperation.

As can be seen in **Figure 3A**, the distance between the United States and the China is closer, the number of common references cited by the research on behalf of the United States and the China is more, and the research topics may be similar; In terms of cooperation between institutions, those in each country still prefer to cooperate with their own institutions. There is close cooperation between Shanghai Jiao Tong University, Huazhong University of Science and Technology, and Chinese Academy of Sciences (**Figure 3C**). International exchange cooperation is still not very close, as shown in **Figure 3C**. Enhanced international collaboration may further promote PTM research in tumor. **Table 1** summarizes the top 15 organizations with the highest contributions. The National Cancer Institute (NCI) had the highest number of publications with 70 papers. The Centre National de la Recherche Scientifique (CNRS) and the Chinese Academy of Sciences (CAS) ranked second and third, with 56 and 50 papers, respectively. Most of the top 15 institutions belong to the United States. A co-cited author refers to two or more authors who are cited together. A network graph of co-cited authors can provide information about influential research groups and potential collaborators, as shown in **Figure 3B**, different colors represent clusters with close cooperation. For example, Chou KC has a high output of articles and has extensive contacts with other authors, especially Xu Y, Chen Z, Chen C, etc.

**Table 1:** The top 15 institutions involved in PTMs of cancer field.

Rank	Institutions	Country	Count
1	National Cancer Institute	United States	70
2	Centre National de la Recherche Scientifique	France	56
3	Chinese Academy of Sciences	China	50
4	The University of Texas MD Anderson Cancer Center	United States	39
5	Johns Hopkins University	United States	39
6	Baylor College of Medicine	United States	33
7	University of Pennsylvania	United States	33
8	Karolinska Institute	Sweden	32
9	University of Oxford	United Kingdom	32
10	University of Michigan	United States	31
11	Memorial Sloan Kettering Cancer Center	United States	30
12	Harvard University	United States	30
13	New York University	United States	27
14	Northwestern University	United States	26
15	University of Toronto	Canada	26

Source: authors with CiteSpace.

**Figure 4:**
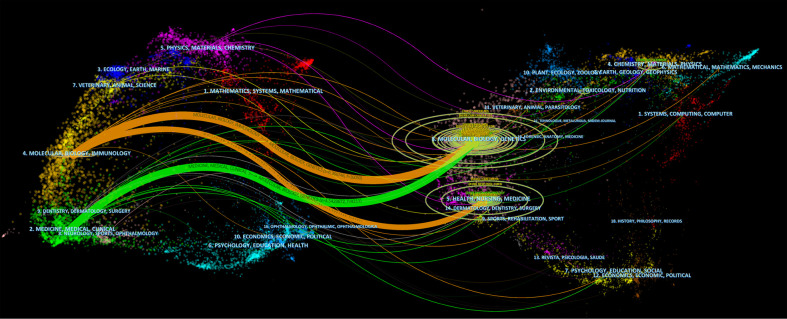
The dual-map overlay of journals. Source: authors with CiteSpace. The left side is the citing journal and the right side is the cited journal. The colored path indicates the cited relationship.

The top 15 highly cited journals are shown in **Table 2**, with the Journal of Biological Chemistry having the highest total citations of 3022. According to the Journal Citation Report (JCR) 2021, 11 of the top 15 journals listed in **Table 2** are in Q1. In **Figure 4**, the two paths in orange are indicated that Molecular/Biology/Immunology journals frequently quoted Molecular/Biology/Genetics journals and Health/Nursing/Medicine journals. Green path is indicated that Medical/Medicine/Clinical journals frequently quoted Molecular/Biology/Genetics journals.

**Table 2:** The top 20 Journals involved in PTMs of cancer field.

Rank	Journals	Country	IF (2021)	JCR	Cited Counts
1	*Journal of Biological Chemistry*	United States	5.157	Q2	3022
2	*Proceedings of the National Academy of Sciences of The United States Of America*	United States	11.205	Q1	2975
3	*Nature*	United Kingdom	49.962	Q1	2748
4	*Cell*	United States	41.582	Q1	2659
5	*Science*	United States	47.728	Q1	2411
6	*Cancer Research*	United States	12.701	Q1	2225
7	*Oncogene*	United Kingdom	9.867	Q1	2039
8	*Journal of Molecular Cell Biology*	China	6.216	Q4	1915
9	*Molecular Cell*	United States	17.970	Q1	1866
10	*EMBO Journal*	United States	11.598	Q1	1788
11	*PloS One*	United States	3.240	Q2	1690
12	*Nucleic Acids Research*	United Kingdom	16.971	Q1	1557
13	*Genes and Development*	United States	11.361	Q1	1516
14	*Biochemical and Biophysical Research Communications*	United States	3.575	Q2	1483
15	*Nature Reviews Cancer*	United Kingdom	60.716	Q1	1395

Source: authors with CiteSpace.

### 3.3 Knowledge-based analysis of PTMs in the cancer field

#### 3.3.1 Analysis of citation path evolution

**Figure 5:**
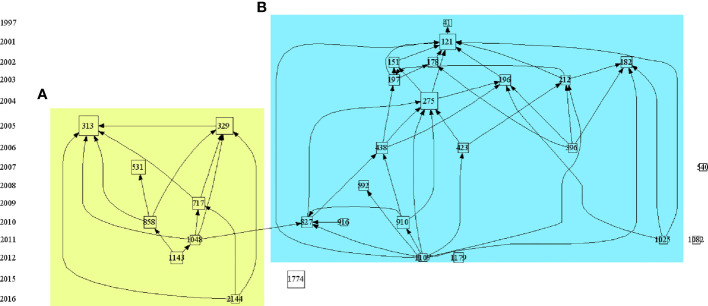
The citation path evolution of PTMs in the cancer field. Source: authors with HistCite. Each node represented one highly cited paper. If paper B cites paper A, there is a line between paper A and paper B, and this line’s arrow pointed to paper B. The size of each node was proportional to the LCS of this paper, larger the node area, the higher the LCS. The yellow background **(A)** represents histone PTMs and the blue background **(B)** represents non-histone PTMs Publication information corresponding to the node was listed in **Table 3**.

By analyzing the citation networks of highly cited papers, we can understand the historical evolution of the field during this period. As shown in **Figure 5**, we selected the 30 most cited papers in the field to build the citation network. Specific literature information is shown in **Table 3**. According to the topics and contents of the papers, these 30 highly cited papers can be divided into two relatively independent parts, representing two research topics.

**Table 3:** The top 30 high-cited papers PTMs of cancer field during 2011 to 2020.

Number	Title	First author	Publication year	Journal	LCS	GCS
41	DNA damage induces phosphorylation of the amino terminus of p53	Siliciano JD	1997	*GENE DEV*	13	688
121	Post-translational modifications and activation of p53 by genotoxic stresses	Appella E	2001	*EUR J BIOCHEM*	59	865
151	Regulation of p53 activity by its interaction with homeodomain interacting protein kinase-2	Hofmann TG	2002	*NAT CELL BIOL*	24	478
178	MDM2 ± HDAC1-mediated deacetylation of p53 is required for its degradation	Ito A	2002	*EMBO J*	22	419
182	Acetylation of p53 Inhibits Its Ubiquitination by Mdm2	Li MY	2002	*J BIOL CHEM*	28	362
196	Pirh2, a p53-Induced Ubiquitin-Protein Ligase, Promotes p53 Degradation	Leng RP	2003	*CELL*	28	549
197	Ubiquitination, phosphorylation and acetylation: the molecular basis for p53 regulation	Brooks CL	2003	*CURR OPIN CELL BIOL*	25	591
212	Phosphorylation Site Interdependence of Human p53 Post-translational Modifications in Response to Stress	Saito S	2003	*J BIOL CHEM*	21	187
275	Post-translational modification of p53 in tumorigenesis	Bode AM	2004	*NAT REV CANCER*	67	948
313	Loss of acetylation at Lys16 and trimethylation at Lys20 of histone H4 is a common hallmark of human cancer	Fraga MF	2005	*NAT GENET*	88	1277
329	Global histone modification patterns predict risk of prostate cancer recurrence	Seligson DB	2005	*NATURE*	67	763
396	The complexity of p53 stabilization and activation	Lavin MF	2006	*CELL DEATH DIFFER*	18	504
423	Modification of p53 with O-linked N-acetylglucosamine regulates p53 activity and stability	Yang WH	2006	*NAT CELL BIOL*	22	306
438	Regulating the p53 pathway: *in vitro* hypotheses, *in vivo* veritas	Toledo F	2006	*NAT REV CANCER*	29	1007
531	How chromatin-binding modules interpret histone modifications: lessons from professional pocket pickers	Taverna SD	2007	*NAT STRUCT MOL BIOL*	44	1079
540	Structure, dynamics and functions of promyelocytic leukaemia nuclear bodies	Bernardi R	2007	*NAT REV MOL CELL BIO*	14	663
592	The FoxO code	Calnan DR	2008	*ONCOGENE*	22	853
717	Global Histone Modifications in Breast Cancer Correlate with Tumor Phenotypes, Prognostic Factors, and Patient Outcome	Elsheikh SE	2009	*CANCER RES*	34	280
827	G9a and Glp Methylate Lysine 373 in the Tumor Suppressor p53	Huang J	2010	*J BIOL CHEM*	28	272
858	Covalent histone modifications — miswritten, misinterpreted and mis-erased in human cancers	Chi P	2010	*NAT REV CANCER*	34	774
910	p53 post-translational modification: deregulated in tumorigenesis	Dai C	2010	*TRENDS MOL MED*	35	355
916	Covalent histone modifications — miswritten, misinterpreted and miserased in human cancers	Saddic LA	2010	*J BIOL CHEM*	14	146
1025	The impact of acetylation and deacetylation on the p53 pathway	Brooks CL	2011	*PROTEIN CELL*	15	199
1048	Histone onco-modifications	Fullgrabe J	2011	*ONCOGENE*	22	190
1082	Targeting protein prenylation for cancer therapy	Berndt N	2011	*NAT REV CANCER*	15	390
1107	Global Histone Modifications in Breast Cancer Correlate with Tumor Phenotypes, Prognostic Factors, and Patient Outcome	Gu B	2012	*INT J BIOL SCI*	15	142
1143	Driver mutations in histone H3.3 and chromatin remodelling genes in paediatric glioblastoma	Schwartzentruber J	2012	*NATURE*	34	1501
1179	The functions and regulation of the PTEN tumour suppressor	Song MS	2012	*NAT REV MOL CELL BIO*	23	1319
1774	PhosphoSitePlus, 2014: mutations, PTMs and recalibrations	Hornbeck PV	2015	*NUCLEIC ACIDS RES*	68	1380
2144	The world of protein acetylation	Drazic A	2016	*BBA-PROTEINS PROTEOM*	17	331

Source: authors with HistCite.

The number correspond to the number in **Figure 5**.

**Figure 6:**
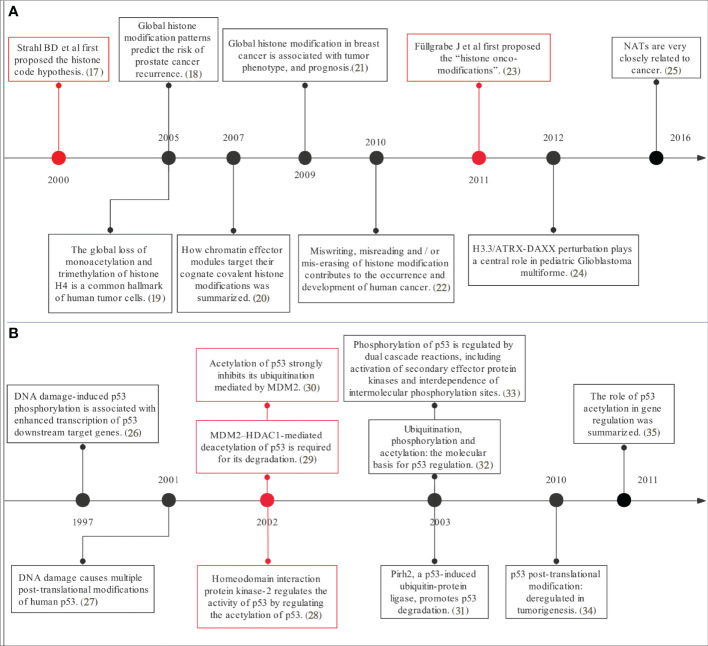
The timeline plot of the citation. Source: authors with ProcessOn (https://www.processon.com). The content in this figure corresponds to **Figure 5** for **(A)** histone modification and tumor study and for **(B)** p53 modification and tumor study.

On the left, the yellow background shows the study of histones PTMs and tumors over the past few years, focusing on the relationship between histone modifications and tumor development and clinical prognosis and their related mechanisms (17–25), linking epigenetics to tumors, with the key event nodes shown in **Figure 6A**.

On the right, the blue background shows the study of non-histone PTMs and tumors over the past few years. Analysis of the top 30 highly cited studies in PTMs and tumor research shows that there are about two-thirds of studies on p53, mostly taking place a decade ago (26–35), indicating that p53 has been widely followed and studied a decade ago, but the mechanism of the post-translational modification of p53 on tumor occurrence, development and prognosis still needs to be further verified. High-cited studies concerning post-translational modifications of p53 with tumors are summarized in **Figure 6B**.

Other studies on tumor and post-translational modifications have focused on the Phosphatase and tensin homolog deleted from chromosome 10 (PTEN). PTEN is a major negative regulator of signaling pathways defined by class I phosphatidylinositol 3 kinase, AKT, and the mechanistic target of rapamycin (mTOR), which also plays a key role in controlling a range of important cellular processes including cell proliferation, growth, survival, and metabolism (36–39). PTEN is regulated by various PTMs, for example, phosphorylation, acetylation, and ubiquitylation. Phosphorylation of PTEN at Ser229, Thr321, Tyr336, Thr366, Ser370, Ser380, Thr382, Thr383, and Ser385 has been shown to be associated with the regulation of tumor suppressor function, cell membrane binding, and stability of PTEN (40). Furthermore, PCAF promotes PTEN acetylation at Lys125 and Lys128, whereas CBP acetylates Lys402. Thioredoxin-interacting protein and peroxiredoxin 1 have been shown to prevent acetylation-mediated PTEN inactivation. The disulfide bond formed between Cys124 and Cys71 by oxidation also reduces the catalytic activity of PTEN, however, this can be avoided by deacetylase sirtuin 1. Ubiquitination of PTEN at Lys13 and Lys289 regulates its tumor suppressor function, subcellular distribution, and stability (40).

#### 3.3.2 Analysis of citations and keywords cluster

**Figure 7:**
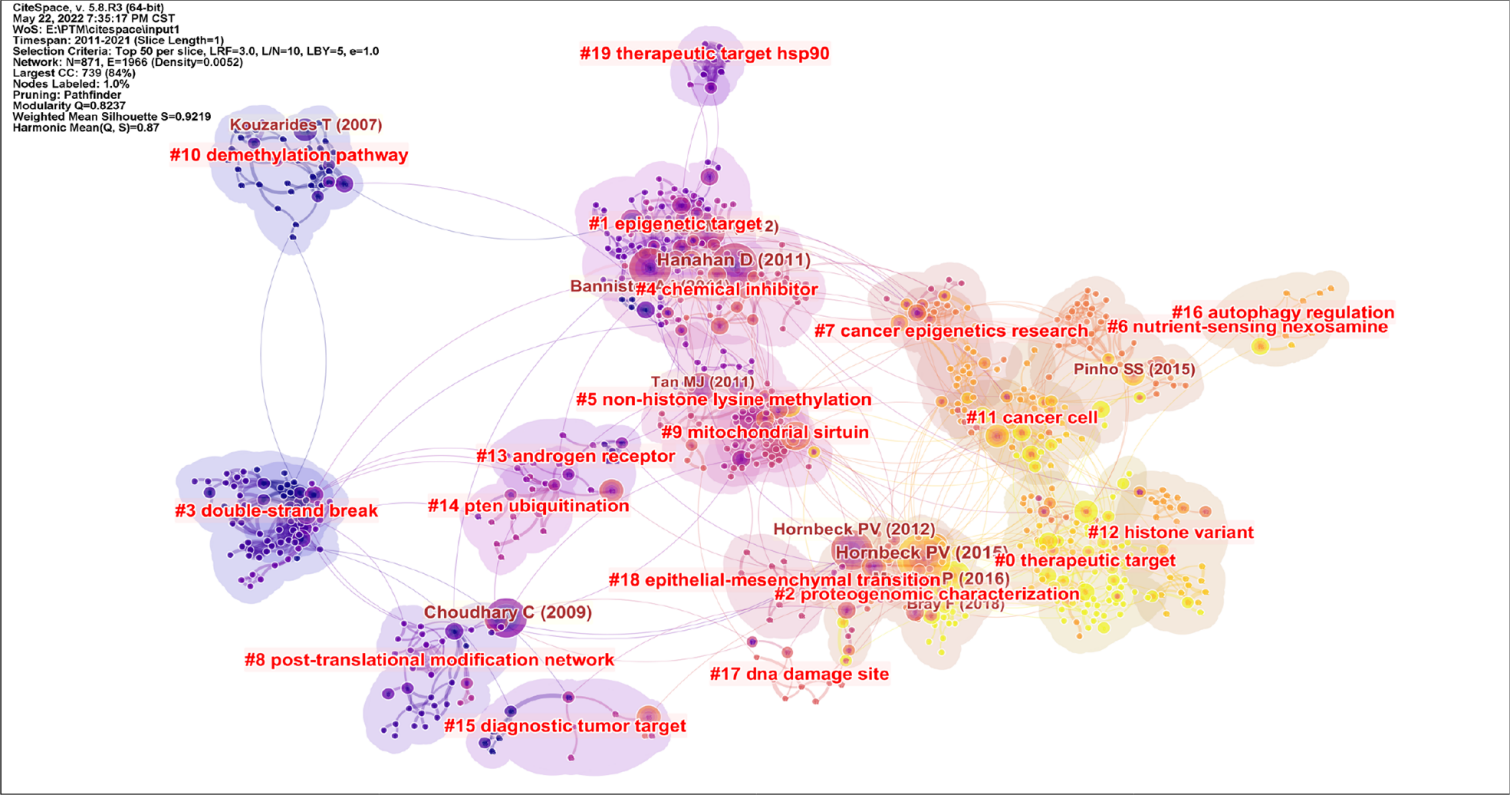
Cluster plot of the co-cited references. Source: authors with CiteSpace. Different colors represent different clusters and each cluster was composed of several closely related studies. The order is 0-18. The smaller the number, the more literature is contained within the cluster. Modularity indicates the modularity of clustering, and the closer it is to 1, the better the clustering result of the network. Silhouette value is used to measure the homogeneity of the network. The closer it is to 1, the higher the homogeneity of the cluster is.

Through the analysis of citations and keywords, we can identify development trends and research hotspots in the research field. The citation clustering diagram is shown in **Figure 7**. As can be seen from the picture, in recent years, scholars have mainly focused on the following aspects. #0 therapeutic target, #1 epigenetic target, #2 proteogenomic characterization, #3 double-strand break, #4 chemical inhibitor, #5 non-histone lysine methylation, #6 nutrient-sensing nexosamine, #7 cancer epigenetics research, #8 post-translational modification network, #9 mitochondrial sirtuin, #10 demethylation pathway, #11 cancer cell, #12 histone variant, #13 androgen receptor, #14 PTEN ubiquitination, #15 diagnostic tumor target, #16 autophagy regulation, #17 DNA damage site, #18 epithelial-mesenchymal transition, and #19 therapeutic target hsp90.

**Figure 8:**
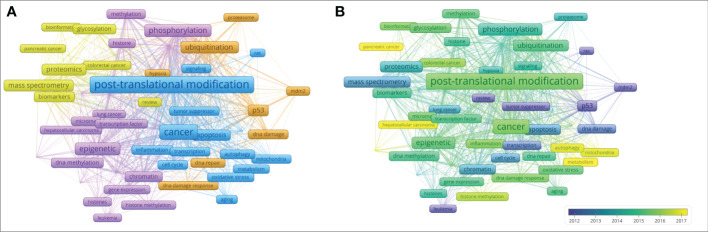
Keyword cluster plot and sequence diagram. Source: authors with VOSviewer. **(A)** Keyword cluster plot generated by VOSviewer. Each color represents a cluster, and clustering is composed of keywords or terms with high co-occurrence frequency. Each node represents a keyword. The higher the frequency, the larger the font. The attachment in the graph represents the connection of two keywords at least once in the literature; the higher the frequency of the keywords, the thicker the line. **(B)** Keyword sequence diagram. The color of the keyword corresponds to the time on the lower right. The closer the color is to yellow, the closer the popular time of the keyword is to the present.

A total of 61 keywords were high-frequency keywords. The clustering diagram is shown in **Figure 8A**. Searching for keywords with long distances and large points may provide new ideas for research in this field. Keywords in each cluster were listed in **Table 4**, and themes were summarized, which were divided into four categories: #1 mechanism, #2 histone, #3 P53, and #4 Key Technologies.

**Table 4:** Clustering of keywords for the study of PTMs in cancer field.

Cluster	Topic	Keywords
#1	Mechanism	post-translational modification, cancer, Aging, apoptosis, autophagy, cell cycle, mitochondria, oxidative stress, ras, tumor suppressor, signaling, inflammation, metabolism, transcription
#2	histone	chromatin, epigenetic, hepatocellular carcinoma, histone, histone methylation, gene expression, methylation, phosphorylation, lung cancer, leukemia, microrna
#3	P53	DNA damage, DNA damage response, DNA repair, hypoxia, mdm2, p53, proteasome, ubiquitination
#4	Key Technologies	biomarkers, bioinformation, colorectal cancer, glycosylation, mass spectrometry, pancreatic cancer, proteomics, review

Source: authors with VOSviewer.

The keywords in blue in **Figure 8A** are the first category of keywords, which mainly describe the mechanism by which PTMs are involved in tumorigenesis and development. PTMs play important roles in regulating protein activity, stability, and folding by inducing covalent attachment of new functional groups such as phosphate, methyl, and acetyl groups to proteins (41). Traditional PTMs, such as phosphorylation and polyurethane, and non-traditional PTMs, such as methylation and acetylation, have been shown to influence the inflammatory response in the cancer field by targeting natural sensors and downstream signaling molecules, including receptors, ligands, enzymes, and transcription factors (42–44). In addition, PTMs can improve the stability of complex signaling pathways through a variety of regulatory mechanisms (38). Although PTMs are closely associated with tumorigenesis, proliferation, and metastasis (1, 45), the molecular mechanisms remain poorly understood. The following examples describe the mechanisms of two kinds of PTMs involved in tumorigenesis and development.

Lysine acetylation is a conserved PTM that links acetyl coenzyme A metabolism to cellular signaling and regulates numerous biological processes by modulating protein interactions, activity, and localization (46). It preferentially targets macromolecular complexes involved in a variety of cellular processes, such as chromatin remodeling, cell cycle, splicing, nuclear translocation, and actin nucleation (47). Glycan drives multiple biological processes in cancer, such as cell signaling and communication, tumor cell dissociation and invasion, cell-matrix interactions, tumor angiogenesis, immune regulation, and metastasis formation, which provide a specific set of targets for therapeutic intervention in the cancer field (48).

Lysine βyhydroxybutyrylation represents a novel acylation modification of proteins mediated by trihydroxybutyric acid, which was first reported by Professor Zhao’s group at the University of Chicago in 2016. They showed that it is closely related to the regulation of fatty acid oxidative metabolism and energy metabolism, and is involved in numerous biological processes such as tumorigenesis and DNA damage repair processes (49). Additionally, lysine 2-hydroxyisobutyrylation (Khib), a PTM of lysine residues, was discovered in 2014 and represents a highly abundant type of PTM that is widely observed in prokaryotes and eukaryotes (50). Khib occurs on histones and plays an important regulatory role in germ cell differentiation. In addition, Khib is closely related to glucose metabolism, amino acid synthesis, glycolysis, and other biological processes. Khib modifications and their regulation are therefore becoming a hot research topic in the field of epigenetics and metabolic regulation (51, 52).

The second type of keyword, marked in purple in **Figure 8A**, mainly describes the relationship between histone modification and tumors. The keywords marked in brown in **Figure 8A** mainly describe the relationship between p53 modification and tumors. The results of keyword cluster analysis and citation path analysis show that the past studies on PTM and tumors are mainly focused on histones and p53, see sub-section on “Analysis of citation path evolution” for details.

The keywords in yellow in **Figure 8A** mainly describe the key technologies and databases in the research of PTMs and cancer. Methodologically, the advent of high-resolution mass spectrometry has greatly facilitated the development of proteomics. In 2011, Baylin proposed a comprehensive proteomic PTM analysis using sequence enrichment and reported a mass spectrometry-based method for the comprehensive analysis of protein expression, phosphorylation, ubiquitination, and acetylation through a series of enrichments for different PTMs in the same biological sample. This technique can be used to quantify nearly 8,000 proteins and over 20,000 phosphorylation, 5,000 ubiquitination, and 3,000 acetylation sites per experiment; thus, generating a holistic view of cellular signaling pathways and can be universally applied to any biological model system and PTM (53). Sharma et al. developed a rigorous experimental and computational workflow capable of mapping over 50,000 different phosphorylated peptides in a single human cancer cell line. Their research showed that phospho-Tyr (P-Tyr) is a functionally independent PTM in the eukaryotic proteome, and the low occupancy of P-Tyr loci observed in unstimulated cells is related to a specific cellular control mechanism (54). Next-generation sequencing links epigenetic abnormalities to mutations that control DNA methylation, DNA packaging, and function in chromatin and metabolism (55). This work has contributed to the understanding of the temporal and spatial regulatory roles of post-translational modifications involved in signaling pathways and networks. The investigators’ work on experimental identification will propel PTM research to a new stage.

At present, the development of PTM-related databases is also gradually becoming systematic. This is because, in addition to the previously identified PTMs, new types of PTMs are constantly being discovered, and the relationship and mechanism between various types of PTMs and various tumors are also being explored. The complex relationship between various PTMs and tumors poses a severe test for researchers to search and explore. In the past few years, experts from various countries have developed databases with their own strengths, which provide a great reference value for future researchers. Cuckoo (http://www.biocuckoo.org/index.php) is a working group developed by Yuxue et al. that includes predictions, tools, and databases. They also provide a detailed categorization summarizing the 233 databases and computational tools that have been developed to date (http://www.biocuckoo.org/link.php). http://dbPTM.mbc.nctu.edu.tw/ and http://ptmcode.embl.de which can predict potential PTM targets can be used to analyze PTM crosstalk and assess PTM-related diseases. MaxQB database (http://maxqb.) can be used to analyze phosphorylation or other PTMs (48). The UniProt database (http://www.uniprot.org) provides a comprehensive, high-quality, and freely accessible resource of protein sequence and functional information (56). The scientific community can also access information on each acetylation site, such as evolutionarily conserved sequences and local secondary structure predictions for proteins surrounding the acetylation site by Phosida (www.phosida.com). The TP53 database (www.iarc.fr/p53/homepage.htm) compiles various types of data and information from the literature and databases on human TP53 gene variants associated with cancer, which is managed by the National Cancer Institute. Phospho Site Plus (http://www.phosphosite.org) is an open, comprehensive, manually managed, and interactive resource for the study of experimentally observed PTMs, primarily in human and mouse proteins. It contains 130,000 non-redundant modification sites, including phosphorylation, ubiquitination, and acetylation sites. The interface is designed for easy navigation and directly from the homepage, users can initiate simple or complex searches and browse high-throughput datasets using disease, tissue, or cell lines (53).

#### 3.3.3 Analysis of new topics of PTMs in the cancer field

Keyword co-occurrence analysis is one of the effective methods to classify research topics. The burst detection of keywords is usually used to select new topics in specific research areas (13). The keywords that have exploded in recent years are obtained through the CiteSpace software, as shown in **Table 5**, where the length of the entire row (red and blue bands) in the last column represents the research period (1997–2022), and the red belt represents the outbreak period. Combining the burst section of the keywords shown in **Table 5** and the keyword sequence diagram shown in **Figure 8B**, we can analyze that the new research keywords in recent years are mainly as follows, protein-protein interaction, epithelial-mesenchymal transition, metabolism, self-renewal, mechanism, receptor, recognition, histone ubiquitination, autophagy, and mitochondria. Scholars also began to pay attention to the application of PTMs in the therapy of tumors where hepatocellular carcinoma and pancreatic cancer have been concerned in recent years. These emerging topics not only help us to understand the frontiers of PTMs in cancer research, but also provide a new source of inspiration for future research to put forward new research questions, scientific hypotheses, and viewpoints.

**Table 5:** The top 30 Keywords with the Strongest Citation Bursts.

Keywords	Stre-ngth	Begin-End	1997 - 2022
messenger rna	5.5	1997-2005	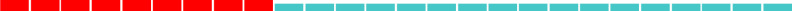
tumor suppressor protein	7.37	2000-2012	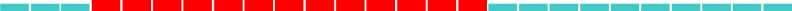
mass spectrometry	8.39	2001-2011	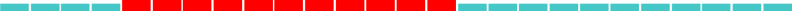
signal transduction	6.15	2001-2006	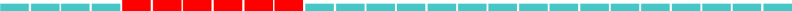
farnesyltransferase inhibitor	5.05	2001-2008	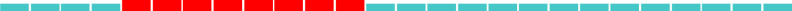
c terminal domain	5.00	2002-2010	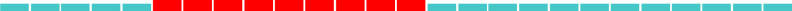
endothelial growth factor	6.23	2003-2011	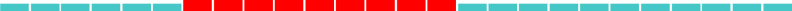
endoplasmic reticulum	5.83	2004-2011	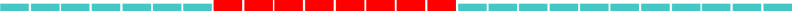
dna binding	5.17	2004-2009	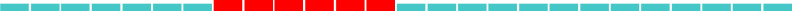
wild type p53	5.01	2004-2009	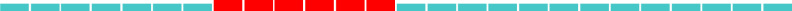
ionizing radiation	7.97	2005-2011	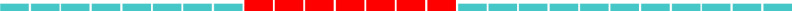
histone deacetylase inhibitor	6.51	2006-2012	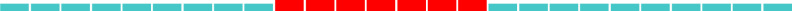
prostate cancer cell	5.51	2007-2012	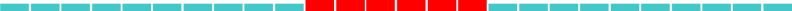
cell cycle arrest	8.25	2008-2015	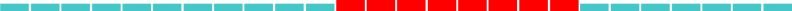
transcriptional activity	6.95	2009-2015	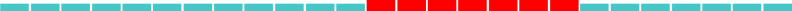
quantitative proteomics	7.76	2010-2012	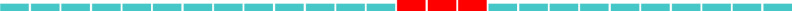
RNA polymerase II	5.31	2011-2015	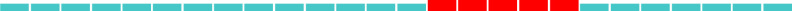
ubiquitin	5.08	2012-2019	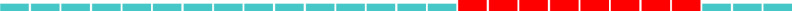
chromatin	5.54	2013-2017	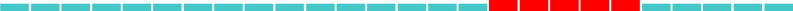
gastric cancer	5.07	2015-2018	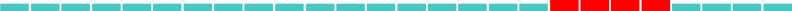
protein phosphorylation	7.14	2016-2020	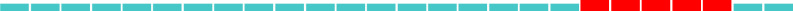
stress	5.35	2016-2020	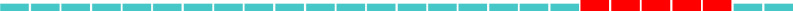
protein-protein interaction	5.15	2017-2019	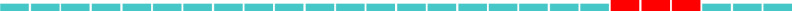
therapy	5.04	2017-2018	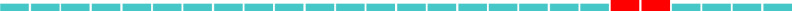
epithelial mesenchymal transition	7.88	2018-2020	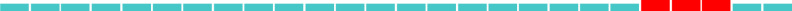
self renewal	5.57	2018-2020	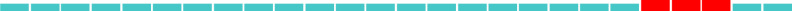
metabolism	5.3	2018-2022	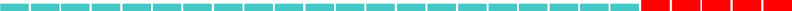
mechanism	6.52	2019-2022	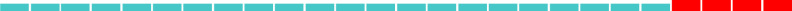
recognition	5.73	2019-2022	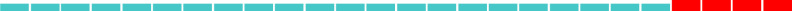
receptor	5.02	2019-2020	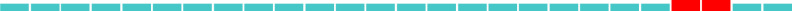

Source: authors with CiteSpace.

### 3.4 Advantages and limitations

This research has several unique advantages. First of all, for the first time, we systematically analyzed the publications related to tumor and post-translational modification by using the method of bibliometrics, which can comprehensively guide scholars who focus on this field. Secondly, we give full play to the advantages of these three softwares (CiteSpace, VOSviewer, and HistCite), and make quantitative and qualitative analysis of the literature in this field, so that the data analysis process is objective and credible. Last but not least, compared with traditional reviews, bibliometrics analysis has unearthed the internal relations among literatures and potential emerging themes in this field, and presented them to readers in a visual way, so that readers can better understand the developing research focus and trends, which undoubtedly laid a foundation for scholars to quickly understand the historical context, knowledge base and development trend of this field. Although it has stability and objectivity, it also has its limitations. In order to meet the requirements of data analysis, we only use English literature published in the WOSCC database. Therefore, the inclusion of papers published in other languages from other databases may increase the rigor of the research.

## 4 Conclusion

There is no doubt that through high-quality research in the past 25 years, we have made great progress in our understanding of post-translational modifications. With the help of information visualization, we can determine the research focus and overall trend in this field, and provide reference information to future researchers. The rapid development of mass spectrometry and the establishment of numerous databases have greatly facilitated the development of proteomics and research on PTMs in the cancer field. Previous studies have mainly focused on histone and p53 modifications. In recent years, researchers have shifted from the study of a single PTM type to the interaction between multiple protein modifications, and paid attention to tumor self-renewal, looking for new receptors and targets. New therapeutic targets in PTMs may be the focus of future research.

## Data availability statement

The original contributions presented in the study are included in the article/**Supplementary Material**. Further inquiries can be directed to the corresponding author.

## Author contributions

RM and ZB conceived and designed the structure of this manuscript. MZ and RM wrote the original draft. YM collected the data. JL and JX performed the analysis. JT and ZM proofread all data and figures. BH, TC, and ZB revised the paper. All authors contributed to the article and approved the submitted version.

## Funding

This work was supported by grants from the National Natural Science Fund of China (82060666, 81702326); the Nature Science Foundation of Gansu Province (21JR7RA354); the Fundamental Research Funds for the Central Universities (lzujbky-2021-ey06).

## Acknowledgments

We would like to thank Editage (www.editage.cn) for English language editing. We also thank the technical support from Jingjie PTM Biolab Co Ltd (Hangzhou, China).

## Conflict of interest

The authors declare that the research was conducted in the absence of any commercial or financial relationships that could be construed as a potential conflict of interest.

## Publisher’s note

All claims expressed in this article are solely those of the authors and do not necessarily represent those of their affiliated organizations, or those of the publisher, the editors and the reviewers. Any product that may be evaluated in this article, or claim that may be made by its manufacturer, is not guaranteed or endorsed by the publisher.
