# Supplementary material for: The Global research of protein post-translational modifications in the cancer field: A bibliometric and visualized study
Source: Front Oncol. 2022 Nov 3;12:978464. doi: 10.3389/fonc.2022.978464 (PMC9670177; doi:10.3389/fonc.2022.978464)
Supplement: Supplementary file 1 [file DataSheet_1.zip › Data Sheet 1.DOC]

label	replace by

cancer-cells	cancer cells

biomarker	biomarkers

breast-cancer	breast cancer

breast-cancer cells	breast cancer

cell-cycle	cell cycle

colorectal-cancer	colorectal cancer

dna-damage	dna damage

epigenetics	epigenetic

gene-expression	gene expression

histone deacetylase inhibitors	histone deacetylase inhibitor

histone modifications	histone modification

inhibitors	inhibitor

mass-spectrometry	mass spectrometry

mechanisms	mechanism

nf-kappa-b	nf-kappa b

pathways	pathway

peptides	peptide

post-translational modification (ptm)	post-translational modification

post-translational modifications	post-translational modification

posttranslational modification	post-translational modification

posttranslational modifications	post-translational modification

Post-translational modifications	post-translational modification

prostate-cancer	prostate cancer

protein-protein interactions	protein-protein interaction

signal-transduction	signal transduction

signaling pathways	signaling pathway

small-molecule inhibitors	small-molecule inhibitor

targets	target

transcription factors	transcription factor

tumor-suppressor genes	tumor-suppressor gene

ubiquitylation	ubiquitination

sumo	sumoylation

ubiquitin	ubiquitination

taiwan	china

microrna	mirna

peoples r china	china

harvard med sch	harvard univ
